# Fluorescent α-Conotoxin [Q1G, ΔR14]LvIB Identifies the Distribution of *α7* Nicotinic Acetylcholine Receptor in the Rat Brain

**DOI:** 10.3390/md22050200

**Published:** 2024-04-27

**Authors:** Hongyu Shan, Nan Wang, Xinyu Gao, Zihan Wang, Jinpeng Yu, Dongting Zhangsun, Xiaopeng Zhu, Sulan Luo

**Affiliations:** 1 Guangxi Key Laboratory of Special Biomedicine, School of Medicine, Guangxi University, Nanning 530004, China; dlhy34@163.com (H.S.); wangnan1801@163.com (N.W.); gxy13518786533@163.com (X.G.); zihanwang0625@163.com (Z.W.); jinpengyu2019@126.com (J.Y.); zhangsundt@163.com (D.Z.); 2 Key Laboratory of Tropical Biological Resources of Ministry of Education, Hainan University, Haikou 570228, China

**Keywords:** α-conotoxins [Q1G, ΔR14]LvΙB, *α7* nAChR, fluorescent peptide, tissue distribution

## Abstract

*α7* nicotinic acetylcholine receptors (nAChRs) are mainly distributed in the central nervous system (CNS), including the hippocampus, striatum, and cortex of the brain. The *α7* nAChR has high Ca^2+^ permeability and can be quickly activated and desensitized, and is closely related to Alzheimer’s disease (AD), epilepsy, schizophrenia, lung cancer, Parkinson’s disease (PD), inflammation, and other diseases. α-conotoxins from marine cone snail venom are typically short, disulfide-rich neuropeptides targeting nAChRs and can distinguish various subtypes, providing vital pharmacological tools for the functional research of nAChRs. [Q1G, ΔR14]LvΙB is a rat *α7* nAChRs selective antagonist, modified from α-conotoxin LvΙB. In this study, we utilized three types of fluorescein after N-Hydroxy succinimide (NHS) activation treatment: 6-TAMRA-SE, Cy3 NHS, and BODIPY-FL NHS, labeling the N-Terminal of [Q1G, ΔR14]LvΙB under weak alkaline conditions, obtaining three fluorescent analogs: LvIB-R, LvIB-C, and LvIB-B, respectively. The potency of [Q1G, ΔR14]LvΙB fluorescent analogs was evaluated at rat *α7* nAChRs expressed in *Xenopus laevis* oocytes. Using a two-electrode voltage clamp (TEVC), the half-maximal inhibitory concentration (IC_50_) values of LvIB-R, LvIB-C, and LvIB-B were 643.3 nM, 298.0 nM, and 186.9 nM, respectively. The stability of cerebrospinal fluid analysis showed that after incubation for 12 h, the retention rates of the three fluorescent analogs were 52.2%, 22.1%, and 0%, respectively. [Q1G, ΔR14]LvΙB fluorescent analogs were applied to explore the distribution of *α7* nAChRs in the hippocampus and striatum of rat brain tissue and it was found that Cy3- and BODIPY FL-labeled [Q1G, ΔR14]LvΙB exhibited better imaging characteristics than 6-TAMARA-. It was also found that *α7* nAChRs are widely distributed in the cerebral cortex and cerebellar lobules. Taking into account potency, imaging, and stability, [Q1G, ΔR14]LvΙB -BODIPY FL is an ideal pharmacological tool to investigate the tissue distribution and function of *α7* nAChRs. Our findings not only provide a foundation for the development of conotoxins as visual pharmacological probes, but also demonstrate the distribution of *α7* nAChRs in the rat brain.

## 1. Introduction

Nicotinic acetylcholine receptors (nAChRs) are widely distributed in human nerve and muscle tissue cells and belong to the Cys-loop superfamily of ligand-gated ion channels (LGICs). According to different tissue distributions, it can be divided into two types: non-muscle nAChRs and muscle nAChRs [1]. The muscle nAChR is located on the postsynaptic membrane of the neuromuscular junction and is a key mediator for the electrical transmission of skeletal muscle tension. Notably, they serve as the target for several clinically used muscle relaxants [2]. Some of the non-muscle nAChR subtypes are located in the presynaptic and postsynaptic regions of cholinergic neurons in the autonomic ganglia and the central nervous system (CNS) and are involved in many processes related to cognitive function, learning and memory, arousal, reward, motor control, and analgesia [3]. The non-muscle nAChRs are pentamers composed of five subunits surrounding the central pore [4], which have permeability to cations such as Na^+^, K^+^, and Ca^2+^. To date, twelve different non-muscle subunits (*α2–α10*, *β2–β4*) have been cloned in vertebrates; among them, the *α8* subunit is only distributed in avian tissues, and the *α7* and *α9* subunits can form homomeric pentamers [5]. Among all various nAChR subtypes, *α4β2* and *α7* are the two major subtypes distributed in the brain [6,7]. *α7* nAChRs are not only expressed in the CNS, but also widely distributed in the peripheral tissues and immune cells, with high Ca^2+^ permeability and the ability to rapidly activate and desensitize [8]. The current investigation revealed that *α7* nAChRs are mainly distributed in the hippocampus [9], striatum [10], cortex [11], and other regions of the brain in the CNS [12], contributing to critical physiological processes such as learning, reward, fear, and cognition. Importantly, *α7* nAChRs dysfunction have been linked to various diseases, including Alzheimer’s disease (AD) [13], epilepsy [14], schizophrenia [15], and Parkinson’s disease (PD) [16]. The presence of *α7* nAChRs in lung cancer tissues suggests a correlation between the receptors and lung cancer [17], and *α7* nAChRs expressed in immune cells such as macrophages play a vital role in cholinergic anti-inflammatory pathways, such as aseptic inflammation [18,19]. Enhancing *α7* nAChR activity can effectively enhance attention and neural cell damage resistance [20], whereas inhibitors targeting *α7* nAChRs have the potential to treat glioblastoma and have certain research and development prospects in clinical applications [21].

The tissue distribution of the *α7* nAChR is closely related to its pharmacological properties, and the issue identification of *α7* nAChRs at the gene level is often limited [22]. Thus, an *α7*-specific visualized fluorescent molecular probe can provide a novel tool for related studies. α-Conotoxins (α-CTxs) are a family of cysteine-rich small neuropeptides (10–25 amino acids) obtained from the venom of marine cone snails [23]. They can selectively bind to nAChRs and differentiate various subtypes [24]. α-CTx, as a pharmacological tool, has high potential in exploring the distribution, physiological, and pathophysiological functions of nAChRs [25,26]. In particular, a series of antagonists selectively targeting rat *α7* nAChRs have been identified from cone snails or α-CTx analogs, such as [V11L, V16A]ArIB (IC_50_: 8 nM) [27], ImI (IC_50_: 191 nM) [28], [A10L]PnIA (IC_50_: 12.6 nM) [29], [A9R, A10L]PnIA (IC_50_: 27 nM) [30], and [Q1G, ΔR14]LvIB (IC_50_: 97 nM) [31].

In addition, a series of fluorescent α-CTxs has been designed and synthesized, thus expanding their application. For example, 5-TAMRA-SE was used to label α-CTx LtΙA, and produced a rat *α3β2* nAChRs selective fluorescent analog [32]. Similarly, the fluorescent molecule Cy5 NHS was used to label α-CTx RgΙA. Fluorochrome-conjugated peptide RgΙA-Cy5 was applied to explore the distribution of the *α9α10* nAChR subtype in RAW264.7 cells [33]. Furthermore, popular fluorophores like BODIPY-FL NHS, Alexa Fluor 488, and BODIPY-FL NHS have been applied to label the CTxs MII, It14a, and [D1G, Δ14Q]LvIC, respectively [34,35]. These fluorescent pharmacological tools facilitate the investigation of the tissue distribution and function of various nAChR subtypes.

In the present study, our laboratory identified a novel α-CTx analog, [Q1G, ΔR14]LvΙB (GCCSNPPCAHEHC*, * indicates C-terminal amidation), derived from α-CTx LvΙB (QCCSNPCAHEHCR*). The potency of [Q1G, ΔR14]LvΙB at inhibiting heterologous rat *α7* nAChRs was better than LvIB: the IC_50_ values were 97 nM and 1760 nM, respectively, an 18-fold increase. Based on [Q1G, ΔR14]LvΙB, we designed and synthesized three fluorescent peptides using three fluorescein molecules: 6-TAMRA-SE, Cy3 NHS ester, and BODIPY-FL NHS ester. Finally, we obtained three fluorescent peptides, namely, [Q1G, ΔR14]LvΙB-6-TAMRA (LvIB-R), [Q1G, ΔR14]LvΙB-Cy3 (LvIB-C), and [Q1G, ΔR14]LvΙB-BODIPY-FL (LvIB-B). The potency, optical characteristics, and stability of LvΙB-R, LvΙB-C, and LvΙB-B were evaluated. These results provide a useful fluorescent molecular probe. By using these tools, we demonstrated that *α7* nAChRs are mainly distributed in the hippocampus, striatum, and other regions of the rat brain.

## 2. Results

### 2.1. Oxidative Folding of α-Conotoxin [Q1G, ΔR14]LvIB

**Figure 1:**
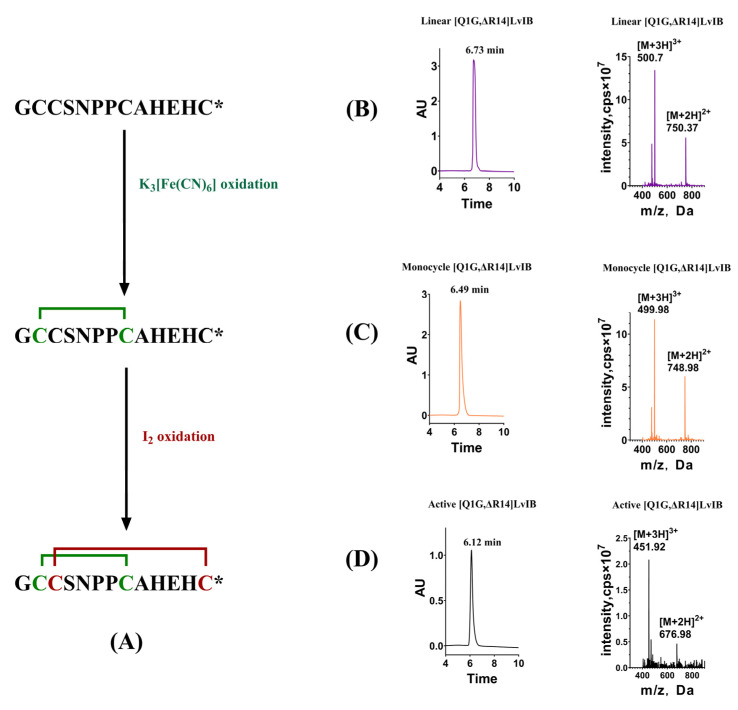
Purification and mass spectrometry identification of [Q1G, ΔR14]LvIB during the oxidative folding process. (**A**) The sequence of [Q1G, ΔR14]LvIB and the disulfide bond formation strategy; (**B**) purification and identification of linear peptides; (**C**) first-step oxidative folding. Purification and identification of monocyclic products; (**D**) second disulfide formed. Purification and identification of final product. All experimental procedures of RP-HPLC with the gradient of 2~70% in 30 min at a flow rate of 12 mL/min. * indicates the C-terminal amide.

To ensure the biological activity of [Q1G, ΔR14]LvIB, the linear peptide was folded in vitro to form the correct disulfide bridge. During the oxidative folding process, the first disulfide bridge was formed in a 20 M K_3_[Fe (CN)_6_] buffer. Next, the Acm protective group was removed in the I_2_ solution and then the second disulfide was formed. The elution time of the final oxidative folding product was 6.12 min. Compared with the linear peptide (elution time 6.73 min), the introduced hydrophobicity exhibited no significant change. The theoretical molecular weights of linear peptides, monocyclic products, and final oxidative folding products were 1498.55 Da, 1496.55 Da, and 1352.49 Da, respectively. Mass spectrometry identified the actual molecular weights of the three compounds as 1499.1 Da, 1496.94 Da, and 1352.76 Da, respectively (Figure 1), consistent with the theoretical values. The purity of the collected products in each step was over 95% and was evaluated by analytical RP-HPLC.

### 2.2. Synthesis of Fluorescent α-Conotoxin [Q1G, ΔR14]LvIB

**Figure 2:**
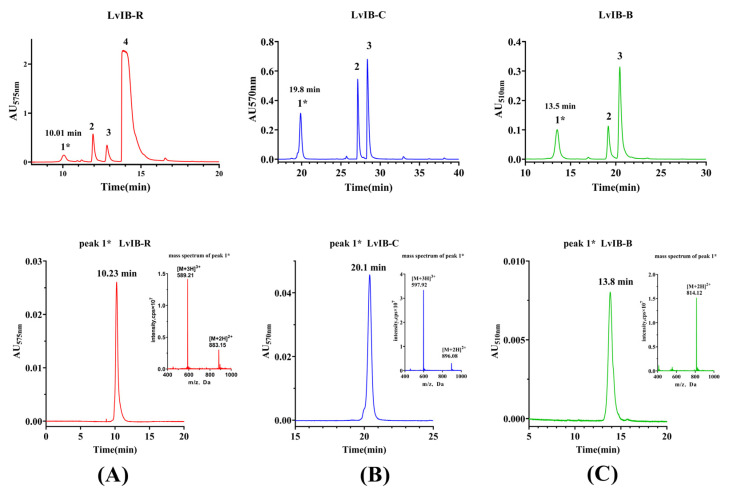
Purification and identification of fluorescent conotoxins. LvIB-R (**A**), LvIB-C (**B**), and LvIB-B (**C**). The peak 1* of the purification procedure represents the fluorescent coupling peptides. The molecular weight of peak 1* was identified by mass spectrometry, which was consistent with the theoretical value. The purification elution gradient was set to 2~20% solution B in 5 min, and 20~70% solution B in 5.01~40 min, at a flow rate of 12 mL/min.

The elution times of the three fluorescent analogs LvIB-R, LvIB-C, and LvIB-B were 10.23 min, 20.10 min, and 13.80 min, respectively (Figure 2). Compared with the native peptide, the hydrophobicity of each fluorescent coupling compound was increased. The purity of all fluorescent peptides was over 95% and identified by analytical RP-HPLC. The theoretical molecular weights of the three fluorescent analogs, LvIB-R, LvIB-C, and LvIB-B, were 1764.94 Da, 1791.50 Da, and 1625.51 Da, respectively. As shown in Figure 2, the actual molecular weights identified by mass spectrometry were 1764.63 Da, 1790.76 Da, and 1626.24 Da, respectively, which was consistent with the calculated values.

### 2.3. Electrophysiological Activity of [Q1G, ΔR14] LvIB and Its Fluorescent Analogs

**Figure 3:**
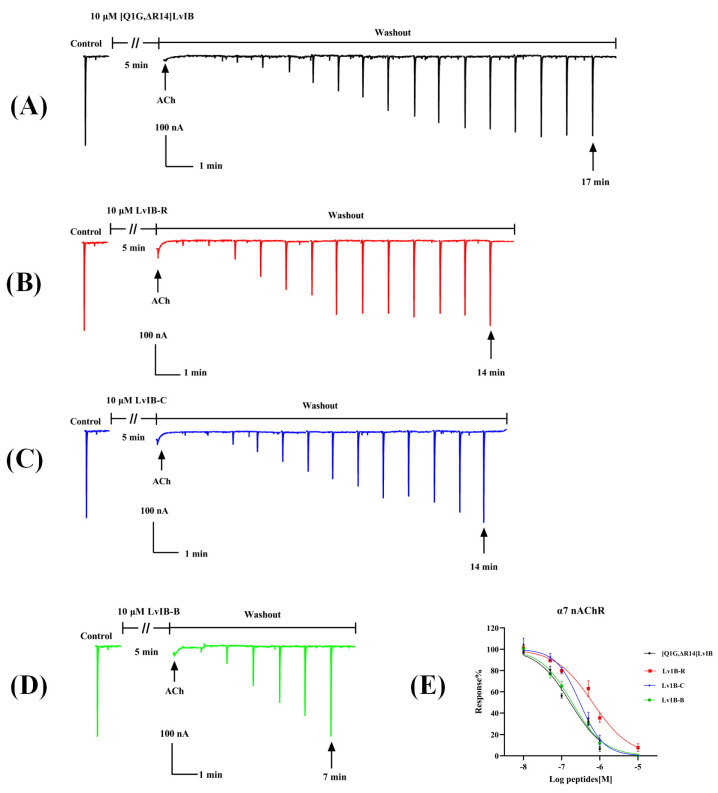
The potency of [Q1G, ΔR14]LvIB fluorescent analogs for rat *α7* nAChRs heterologously expressed in *X. laevis* oocytes. (**A**–**D**) [Q1G, ΔR14]LvIB and three fluorescent analogs display different reversibility after blocking with 10 μM peptides. [Q1G, ΔR14]LvIB (**A**); LvIB-R (**B**); LvIB-C (**C**); LvIB-B (**D**). The control indicates the response to 200 μM ACh. The oocytes were incubated with 10 μM peptides for 5 min followed by the application of ACh. The ND96 flow rate was 2 mL/min. (**E**) The concentration–response curves of [Q1G, ΔR14]LvIB and its fluorescent analogs.

The electrophysiological results revealed that the LvIB-R, LvIB-B, and LvIB-C conjugates exhibited some modifications in terms of their antagonistic effects at ACh-evoked currents mediated by rat *α7* nAChRs heterologously expressed in *X. laevis* oocytes compared to native [Q1G, ΔR14]LvIB. At 10 μM, [Q1G, ΔR14]LvIB demonstrated an inhibition rate of 98 ± 0.12% (*n* = 3), while LvIB-R, LvIB-B, and LvIB-C showed inhibition rates of 87 ± 0.24% (*n* = 3), 96 ± 0.11% (*n* = 3), and 94 ± 0.20% (*n* = 3), respectively. There was no significant difference in the inhibitory effect between [Q1G, ΔR14]LvIB and its fluorescent analogs. Furthermore, the recovery from 10 µM inhibition indicated that the native [Q1G, ΔR14]LvIB had 90% current restoration within 17 min (Figure 3A), and LvIB-R and LvIB-C had a similar affinity with approximately 90% current restoration within 14 min (Figure 3B,C). However, LvIB-B exhibited a faster elution rate with current restoration to ~90% within just 7 min (Figure 3D). To determine the potency of [Q1G, ΔR14]LvIB and its fluorescent analogs, we calculated the IC_50_ values. As shown in Figure 3E, the IC_50_ values of [Q1G, ΔR14]LvIB and its analogs were 165.9 nM, 643.3 nM (LvIB-R), 298.0 nM (LvIB-C), and 186.9 nM (LvIB-B), respectively (Table 1). In comparison to native [Q1G, ΔR14]LvIB, the IC_50_ value of LvIB-R decreased about fourfold. The IC_50_ values of LvIB-B and LvIB-C exhibited no obvious changes in the inhibition of *α7* nAChRs. In particular, the activity of LvIB-B was approximated to [Q1G, ΔR14]LvIB.

In addition, at a 10 μM concentration, the potency of [Q1G, ΔR14]LvIB against *α6*/*α3β4* and *α3β2* nAChR subtypes, the inhibition rate was 88 ± 5% (*n* = 3) and 97 ± 2% (*n* = 3), respectively (Figure S1). However, the electrophysiological assay against rat *α6*/*α3β4* nAChRs demonstrated that both LvIB-C and LvIB-B exhibited inhibitory activity of 10 ± 0.3% (*n* = 3) and 0 ± 2% (*n* = 3) at the concentration of 10 μM, which almost lost pharmacology-inhibiting potency. For rat *α3β2* nAChR subtypes, both LvIB-C and LvIB-B exhibited inhibitory 50 ± 2% (*n* = 3) and 64 ± 4% (*n* = 3) at 10 μM (Figure S1). To identify the potency of [Q1G, ΔR14]LvIB, LvIB-B, and LvIB-C against *α3β2* nAChRs, the IC_50_ values were calculated to 945 nM, 10.92 μM, and 2.55 μM, respectively (Figure S2). Thus, LvIB-B maintained similar potency and selectivity for rat *α7* nAChRs, and it can serve as an ideal pharmacological tool.

### 2.4. Stability Analysis of [Q1G, ΔR14]LvIB Fluorescent Analogs in Artificial Cerebrospinal Fluid

**Figure 4:**
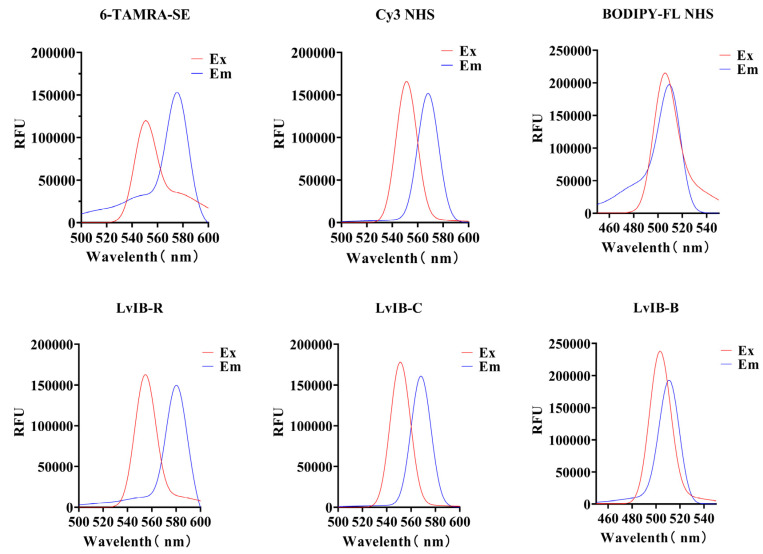
Spectral analysis of fluorescent analogs. Ex represents the excitation wave, Em represents the emission wave, and RFU indicates the relative fluorescence intensity detected by the UV spectrophotometer.

The unique molecular structures and conjugated double bonds of fluorescent dyes 6-TAMRA-SE, Cy3 NHS, and BODIPY-FL NHS provide sensitive fluorescence signals. When [Q1G, ΔR14]LvIB is coupled with different fluorophores, the fluorescence spectrum of the fluorescent analogs should not change significantly compared with the fluorescent dye. The excitation/emission (Ex/Em) values of the fluorescent molecules 6-TAMRA-SE, Cy3 NHS ester, and BODIPY-FL NHS ester were 550 nm/575 nm, 550 nm/570 nm, and 505 nm/510 nm, respectively, and the Ex/Em value of LvIB-R, LvIB-C, and LvIB-B were 555 nm/580 nm, 550 nm/570 nm, and 505 nm/510 nm, respectively. The results indicated that the wave change trend of the fluorescence spectrum was similar to the wave change trend of the fluorescent molecule itself, and the excitation wavelength and emission wavelength were not significantly different. The result is shown in Figure 4.

**Figure 5:**
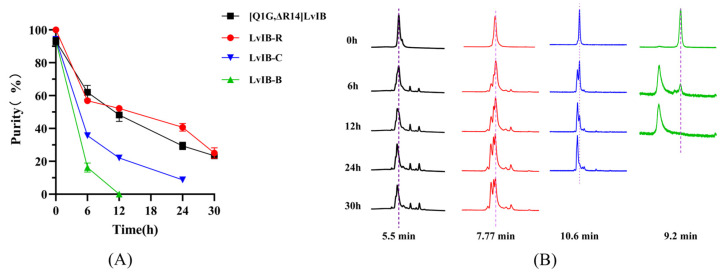
Stability analysis of fluorescent analogs in aCSF. (**A**) The line graph represents the residual degradation rate of [Q1G, ΔR14]LvIB and its fluorescent analogs. (**B**) The main peak degradation process of each peptide at different times.

As an ideal pharmacological tool to explore the distribution of *α7* nAChRs in various brain regions, the fluorescent peptides should have a certain stability in artificial cerebrospinal fluid (aCSF). The stability of LvIB-R, LvIB-B, and LvIB-C in aCSF was analyzed at 37 °C. As shown in Figure 5A, the residual rates of [Q1G, ΔR14]LvIB, LvIB-R, LvIB-C, and LvIB-B at 12 h were 48.2%, 52.2%, 22.1%, and 0%, respectively. Each data point was collected by the average of five individual experiments. Although LvIB-C maintained similar potency with native [Q1G, ΔR14]LvIB, its stability was moderately reduced. As the most unstable in the aCSF, LvIB-B was almost undetectable at the 12th hour. In contrast, LvIB-R maintained consistent stability compared with the native [Q1G, ΔR14]LvIB. The result is shown in Figure 5.

### 2.5. Fluorescence Imaging of Rat Brain Slices

**Figure 6:**
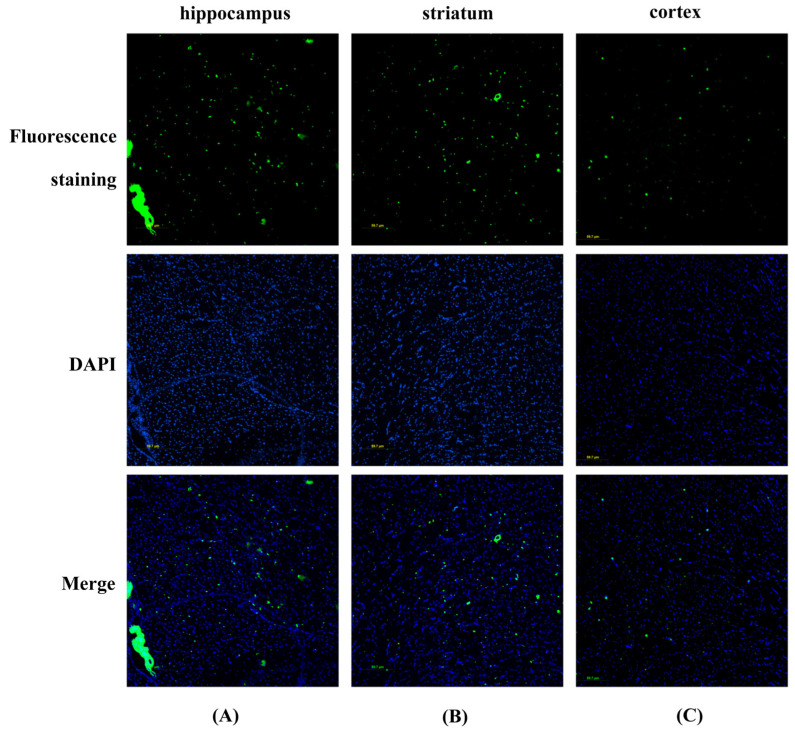
LvIB-B showed obvious fluorescence distribution in the hippocampus, striatum, and cortex in rat brain slices. The top row is the green fluorescence emitted by LvIB-B, the middle row is the blue fluorescence emitted by DAPI after nuclear staining, and the bottom row is the combination of green and blue fluorescence. (**A**) The hippocampus was labeled with LvIB-B to a large number of receptors, showing green fluorescence. (**B**) More fluorescence was also seen in the striatum region. (**C**) Cortical areas are marked with a small amount of *α7* nAChRs, with less distribution in the cortex than in the hippocampus and striatum areas.

To explore the distribution of *α7* nAChRs in different brain regions, rat brain slices were incubated with 1 μM of either LvIB-R, LvIB-C, or LvIB-B, respectively. We observed distinct fluorescence distribution in the hippocampus, striatum, and cortex for all three fluorescent analogs. As shown in Figure 6 and Figures S3 and S4 upon DAPI staining of the nucleus (blue), we noticed the localization of red fluorescent spots (LvIB-R and LvIB-C) on the cell membrane. Interestingly, a greater number of green fluorescent spots (LvIB-B) were also observed on the cell membrane. These results revealed different distributions of *α7* nAChRs in the brain regions and affinity differences among the three analogs. Considering both signal strength and stability, LvIB-B appears to offer the most ideal imaging features (Figure 6). The control group images are shown in Figures S5–S7. Images of other brain regions are displayed in Figure S8.

## 3. Discussion

*α7* nAChRs are vital ligand-gated ion channels (LGIC) mainly distributed in the CNS, and are responsible for a series of vital physiological activities such as learning, reward, and perception. It is crucial to clarify the mechanism of *α7* nAChR-related diseases and explore the targeting drugs. The targets of nAChRs agonists include endogenous acetylcholine (ACh) and exogenous nicotine [36], which can briefly open the channel for a few milliseconds, and then close and return to a resting or desensitized state. Antagonists of *α7* nAChRs include α-bungarotoxin (α-BGT) [37], methyllycaconitine (MLA) [38], and α-CTx. 

α-CTxs are a class of neuropeptide-specific targeting nAChRs, which have advantages such as small molecule size, strong activity, and a stable structure. By conjugating α-CTxs with fluorescent markers, the interaction between conotoxins and their respective receptors can be directly visualized using fluorescence microscopy. To date, some fluorescent peptides have been designed and applied in nAChR-related investigations. For example, the IC_50_ values of LtIA and its fluorescent peptide LtIA-5-TAMRA targeting *α3β2* are 22.95 nM and 90.66 nM, respectively. The activity of LtIA-5-TAMRA is weakened by about fourfold compared to the native LtIA [32]. The IC_50_ values of RgIA and its fluorescent peptide RgIA-Cy5 targeting *α9α10* nAChRs are 1.6 nM and 5 nM, respectively. The IC_50_ values only changed about threefold after modification [33]. Previous results have shown that fluorescent molecules can be conjugated to conotoxin without significantly affecting the potency and selectivity.

Currently, the distribution and function of *α7* nAChRs are still unclear because of the lack of specific and visual pharmacological tools. [Q1G, ΔR14]LvIB, a novel *α7* nAChRs antagonist discovered by our laboratory, has the potential to be further developed into such a visual probe. The fluorescently labeled [Q1G, ΔR14]LvIB provides a useful pharmacological tool to explore the distribution of *α7* nAChRs in different brain regions. Considering the differences in optical properties, fluorescent group size, and structure, 6-TAMRA-SE, Cy3 NHS ester, and BODIPY-FL NHS ester were selected as ideal fluorescent molecules. They can be conjugated with the N-terminus or side-chain Lys residues of peptides after being treated with NHS-activated esters. In this experiment, [Q1G, ΔR14]LvIB without Lys and fluorescent molecules can be connected to the N-terminal of [Q1G, ΔR14]LvIB. Our experiments revealed that the fluorescent labeling of [Q1G, ΔR14]LvIB led to certain changes in its properties. The IC_50_ value identified by the TEVC showed that LvIB-R has an obvious decrease in activity compared to [Q1G, ΔR14]LvIB of about fivefold. It has been confirmed that all three fluorescent α-CTxs have selective targeting properties towards *α7* nAChRs. Compared with their corresponding fluorescent molecules, the optical properties of fluorescent α-CTxs did not show significant changes, and their fluorescence characteristics were relatively stable. According to the fluorescence imaging results, all three fluorescent α-CTxs could target the distribution of *α7* nAChRs in rat brain tissue slices (hippocampus, striatum, and cortex), proving that the visualization of α-CTxs binding with receptors can be achieved under fluorescence microscopy. However, through image observation, there were still differences in the fluorescence staining effect of the three kinds of fluorescein on the tissue slices.

In conclusion, this study successfully developed fluorescently labeled analogs of α-CTx [Q1G, ΔR14]LvIB, enabling the visualization of *α7* nAChRs distribution. These analogs offer an ideal visualization tool for investigating the relationship between *α7* nAChRs localization and function. Considering the superior potency and outstanding imaging characteristics, LvIB-B has potential applications as an ideal pharmacological tool to explore *α7* nAChRs distribution and function. However, the observed instability of LvIB-B in artificial cerebrospinal fluid highlights an area for further optimization in future studies. 

## 4. Materials and Methods

### 4.1. Materials

The crude linear peptide [Q1G, ΔR14]LvΙB (purity was about 80%) was synthesized by Bankpeptide Biological Technology Co., Ltd. (Hefei, China). Potassium ferricyanide (K_3_[Fe(CN)_6_]), iodine (I_2_), ascorbic acid, sodium tetraborate, and trifluoroacetic acid (TFA) (HPLC grade) were purchased from McLean Biochemical Technology Co., Ltd. (Shanghai, China), and acetonitrile (ACN) (HPLC grade) from Fisher Scientific (Pittsburgh, PA, USA). Dimethyl sulfoxide (DMSO) (HPLC grade) was purchased from Sangon Biotech Co., Ltd. (Shanghai, China). Fluorescent dyes 6-TAMRA-SE, Cy3 NHS, and BODIPY-FL NHS were purchased from Yuanye Biotechnology Co., Ltd. (Shanghai, China). Artificial cerebrospinal fluid (aCSF) was purchased from Fuzhou Feijing Biotechnology Co., Ltd. (Fuzhou, China). The Reversed-Phase High-Performance Liquid Chromatography (RP-HPLC) semipreparative C18 Vydac column (130 Å, 5 μm, 19 mm × 100 mm) and analytical C18 Vydac column (100 Å, 5 μm, 4.6 mm × 250 mm) were purchased from Waters Corporation (Shanghai, China). Acetylcholine (ACh), collagenase, and bovine serum albumin (BSA) were purchased from Sigma-Aldrich (St. Louis, MS, USA). Servicebio (Wuhan, China) performed a frozen section experiment on rat brain tissues. 4-6-diamino-2-phenylindole (DAPI), 4% paraformaldehyde (PFA), and phosphate-buffered saline (PBS) were purchased from Servicebio (Wuhan, China). *Xenopus laevis* (*X. laevis*) was obtained from the Kunming Institute of Zoology. *Rattus norvegicus* (Rats) were purchased from the SPF Biotechnology Co., Ltd. (Beijing, China). The procedure for the operation of *X. laevis* and the rat was approved by the Ethics Committee of Guangxi University (GXU-2022-159), and the whole process strictly adhered to the guidelines for the care and use of laboratory animals. Plasmids containing various nAChR subunit genes were kindly provided by Dr. J. Michael McIntosh (The University of Utah).

### 4.2. Oxidative Folding and Purification of α-Conotoxin [Q1G, ΔR14] LvΙB 

[Q1G, ΔR14]LvΙB has an active disulfide bond pattern of [Ⅰ-Ⅲ, Ⅱ-Ⅳ](Cys2-Cys8, Cys3-Cys13). To achieve this connection, Cys types in the linear peptide are protected with different protecting groups: Cys2 and Cys8 are protected by S-triphenylmethyl (Trt), and Cys3 and Cys13 are protected by S-acetyl aminomethyl (Acm). The [Q1G, ΔR14]LvΙB linear peptides were purified by semi-preparative RP-HPLC. The purity of linear peptides is higher than 95% before the oxidative folding procedure. Purified linear peptides were sequentially subjected to K_3_[Fe (CN)_6_] and iodine oxidation, respectively. The detailed procedure was described previously [31]. Briefly, the first step occurred in 20 M K_3_[Fe (CN)_6_] solution, at room temperature (25.0 ± 1.0 °C,) for 50 min. The Trt protective groups were cleaved and the first disulfide bridge formed. Then, the monocyclic products were purified and oxidated in an iodine solution. In 1 nM I_2_ solution, the monocyclic peptides were dropped slowly, kept in the dark, and stirred for 20 min. The second disulfide bridge was formed and the final products were obtained. The products of each step were purified by semi-prepared RP-HPLC (Waters 2489, Milford, MA, USA). Solution A was 0.05% TFA and solution B was 90% acetonitrile. The elution procedure was performed under gradient conditions at a flow rate of 12 mL/min, with solution B varying from 2 to ~70% in 30 min. The purity and molecular weight of these peptides were analyzed by Ultra Performance Liquid Chromatography (UPLC) (Waters ACQUITY UPLC H-Class PLUS, Milford, MA, USA) and mass spectrometry. The purity of all peptides was over 95%. To identify the intermediate and final products, the molecular weight was determined by Electrospray Ionization Mass Spectrometry (ESI-MS) (Waters Xevo TQD, Milford, MA, USA), The quantification of the final product was determined by analytical RP-HPLC (Waters e2695, Milford, MA, USA), and its content was determined based on its standard quantification curve and chromatographic peak properties.

### 4.3. Synthesis and Identification of Fluorescent α-Conotoxin [Q1G, ΔR14] LvΙB

**Figure 7:**
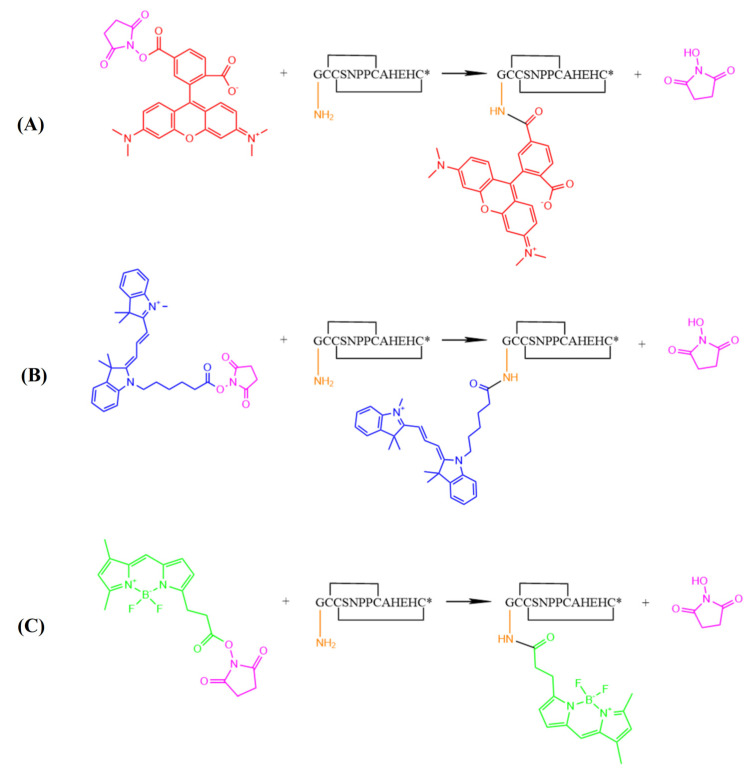
Schematic representation of the fluorescent molecules conjugated with α-CTx [Q1G, ΔR14]LvΙB. (**A**). 6-TAMRA-SE (LvIB-R), (**B**) Cy3 NHS ester (LvIB-C), and (**C**) BODIPY-FL NHS Ester (LvIB-B). * represents C-terminal amidation.

Fluorescent molecules 6-TAMRA-SE, Cy3 NHS ester, BODIPY-FL NHS Ester have an activated structure, NHS [39]. Fluorescent molecules can ligate with the N-terminus or lysine (Lys, K) of the peptide in a weak alkaline condition. Due to [Q1G, ΔR14] LvΙB being without Lys, the fluorescent molecule is connected to the N-terminal of [Q1G, ΔR14] LvΙB, as shown in Figure 7. To conjugate fluorescent molecules with [Q1G, ΔR14] LvΙB, three fluorescent compounds (dissolved in DMSO at a concentration of 15 mM) were added to the above reaction solution under dark conditions. The reaction was shaken at 37 °C for 6 h (6-TAMRA, R), 1h (Cy3, C), and 3h (BDP, B). Finally, 200 μL water and 300 μL DMSO were added to terminate the reaction. The reaction products were purified by RP-HPLC. The conditions for gradient elution were 2%~20% solution B in 5 min, and 20%~70% solution B in 5.01 to 40 min. Based on the optical properties of the different fluorescent groups 6-TAMRA, Cy3, and BODIPY FL, the UV detection wavelengths were adjusted according to the fluorophores: 575 nm (6-TAMRA), 570 nm (Cy3), and 510 nm (BODIPY-FL). The purity was identified by analytical RP-HPLC. All single peaks were collected and identified by mass spectrometry to be consistent with the theoretical molecular weight. The purity of all fluorescent conotoxins used in subsequent experiments was over 95%. The corresponding fluorescent peptides of the three fluoresceins were named LvIB-R (6-TAMRA-SE), LvIB-C (Cy3 NHS), and LvIB-B (BODIPY-FL NHS Ester), respectively.

### 4.4. Electrophysiology

The plasmid containing rat *α7* nAChR cDNA was linearized with *Sma* I, and capped RNA (cRNA) of *α7* nAChR subunits was prepared through in vitro transcription with the mMESSAGE mMACHINE™ T7 Transcription Kit (Thermo Fisher Scientific, Pittsburgh, PA, USA). The detailed process of cRNA preparation was described previously [40]. *X. laevis* oocytes were isolated and then microinjected with a specific volume of cRNA (50.6 nl, ~25 ng/cell). The oocytes were cultured at 17 °C for 2 to 3 days before electrophysiological detection. The potency of conotoxin and fluorescent products was evaluated using a two-electrode voltage clamp (TEVC) [41]. *α7* nAChR subtypes were expressed on the membrane of the oocytes. Quantified samples of [Q1G, ΔR14]LvIB and LvIB-R, LvIB-C, and LvIB-B were dissolved in ND96 solution (96 mM NaCl, 2.0 mM KCl, 1.0 mM MgCl_2_, 1.8 mM CaCl_2_, 5 mM HEPES, 1 μM atropine and 0.1 mg/mL BSA; pH adjusted to 7.5). Different concentrations of peptides (ranging from 10^−8^ to 10^−4^ M) were incubated with oocytes for 5 min to evaluate their potential activity against *α7* nAChRs. The volume of the chamber was approximately 50 μL. The holding potential was clamped at −70 mV. In one sweep, the oocyte was stimulated with 200 μM ACh for 2 s (the agonist ACh EC_50_ value is 281.7 μM for rat *α7* nAChRs); subsequently, the solenoid valve switched to ND96 solution for 58 s at a rate of 2 mL/min. Each trial contained three sweeps. The ACh-induced current was recorded and analyzed with the Digitata 1550B data acquisition system and Clampex software (Molecular Devices Corp., Sunnyvale, CA, USA). The potency of various peptides against *α7* nAChRs were evaluated by half-maximal inhibitory concentration (IC_50_) values. Meanwhile, we also tested the potency of three fluorescent peptides against other nAChR subtypes expressed in *X. laevis* oocytes. For *α3β2* and *α6β4* nAChR subtypes, the channel currents were induced with 100 μM ACh.

### 4.5. Stability Analysis

[Q1G, ΔR14]LvIB and LvIB-R, LvIB-C, and LvIB-B (all 10 μM) were analyzed using a UV spectrophotometer to identify whether the optical properties of the luminescent groups of the dye molecules were affected in the synthesis of fluorescent peptides. To investigate the metabolism of [Q1G, ΔR14]LvIB and its fluorescent peptide in the CNS, cerebrospinal fluid stability analysis was performed. The samples were dissolved in aCSF (the purity of the samples was over 95%) and placed in a metal bath at 37 °C. An equal volume of samples was taken out at 0, 6, 12, 24, and 30 h for analysis by analytical HPLC. The purity of the main peak was determined by analytical HPLC to represent the metabolism of peptide in the cerebrospinal fluid environment, and the changing trend of its composition was recorded. The experiment was repeated five times for each sample, and the final data were averaged. The data were analyzed by Prism 8.0 software (GraphPad Software Inc., San Diego, CA, USA).

### 4.6. Fluorescence Imaging

Ten-week-old rats were sacrificed, and the brain tissues were removed completely, rinsed with sterile PBS, and quickly frozen in liquid nitrogen to ensure the freshness of the tissues. The dissection procedure was approved by the Ethics Committee of Guangxi University, and the guidelines for the use of experimental animals were strictly followed under the condition of ensuring animal welfare. The tissues were prepared by the Servicebio Corporation under low-temperature conditions to prepare frozen brain slices. The three fluorescent peptides were prepared in PBS as a solution of 10^−6^ M to incubate the slices. Coronal and horizontal slices of the rat brains were dried and oven-dried at 37 °C for 30 min to remove the water from the frozen slices. After fixation in 4% PFA for 30 min, the slices were rinsed using PBS for 5 min, which was repeated three times. The fluorescent peptide solution was dropped onto the tissue slices and evenly covered, and the cells were incubated for 5 min. The slices were rinsed again with PBS for 5 min and this was repeated three times. After the slices were dried, they were uniformly covered by dropping DAPI, incubated for 5 min, and repeated three times by rinsing with PBS. After drying the slices, a small amount of anti-fluorescence quench agent was added and the coverslips were gently covered. The excitation wavelength of the fluorescence microscope (Nikon Eclipse Ti 2, Tokyo, Japan) was adjusted to observe the labeling of fluorescent molecules on the tissue slices. The images taken were processed by the software ImageJ 1.54d (ImageJ Software Inc., Bethesda, Maryland, USA).

### 4.7. Data Analysis

Electrophysiological data were analyzed using Clampfit software (Molecular Devices, Sunnyvale, CA, USA), while statistical graphing and curve fitting were performed with GraphPad Prism 8.0 (GraphPad Software Inc., San Diego, CA, USA). In the electrophysiology experiments, the mean peak current values of the three control responses were used to standardize the amplitude of each test response to obtain a “response %”. The dose–response data were fit to the Hill equation: response % = 100/{1 + ([toxin]/IC_50_)^nH^}, where nH is the Hill coefficient. Each data point of the dose–response curve was represented as Mean ± SEM. The IC_50_ values of antagonist against *α7* nAChRs were obtained from at least 6 oocytes and the inhibition for the other nAChR subtypes were recorded for at least 3 oocytes. Significance was determined at 95% confidence intervals, and the oocytes used in the experiment came from at least three batches of *X. laevis*. To identify the fluorescent peptide stability in aCSF, each data point was indicated as Mean ± SEM (*n* = 5). In fluorescence imaging, the rat brain slices were taken from 3 different batches, and no less than 10 imaging photos were taken in each group. 

## Figures and Tables

**Table 1:** IC_50_ and Hill slope values of native [Q1G, ΔR14]LvIB and three fluorescent analogs on *α7* nAChRs.

Peptide	IC_50_ (nM)	Hill Slope
[Q1G, ΔR14]LvIB	165.9 (135.3–203.5)	0.99 (0.8–1.19)
LvIB-R	643.3 (494–837.7)	0.89 (0.67–1.14)
LvIB-C	298 (238.5–372.3)	1.39 (1.05–1.72)
LvIB-B	186.9 (150.1–232.8)	1.04 (0.83–1.24)

IC_50_ values with 95% confidence interval; Hill slope obtained from concentration–response curves for [Q1G, ΔR14]LvIB and its fluorescent analogs at rat *α7* nAChRs. All data were obtained from more than 6 independent oocytes.

## Data Availability

Data will be made available on request.

## Supplementary Materials

The following supporting information can be downloaded at: https://www.mdpi.com/article/10.3390/md22050200/s1, Figure S1: Potency of [Q1G, ΔR14]LvIB, LvIB-C, and LvIB-B against other nAChR subtypes; Figure S2: Concentration-response analysis of [Q1G, ΔR14]LvIB and its fluorescent analogs against *α3β2* nAChRs; Figure S3: LvIB-R showed obvious fluorescence distribution in the hippocampus, striatum, and cortex of rat brain slices; Figure S4: LvIB-C showed obvious fluorescence distribution in the hippocampus, striatum, and cerebral cortex of rats; Figure S5: Competitive inhibition of *α7* nAChRs when 1 μM concentration of native peptide and fluorescent analogs were co-incubated with the hippocampus of rat brain slices; Figure S6: Fluorescence of [Q1G, ΔR14]LvIB and fluorescent analogs at 1 μM concentration in the striatal region of rat brain slices; Figure S7: Fluorescence images of the cortex region in rat brain slices showing the competitive inhibition effect of native peptide and fluorescent analogs at 1 μM concentration; Figure S8: Different staining effects of three fluorescent peptides in the cerebellar region. Some *α7* nAChRs can be labeled by fluorescent analogs and can be used as pharmacological tools.
