# Supplementary material for: Fluorescent α-Conotoxin [Q1G, ΔR14]LvIB Identifies the Distribution of α7 Nicotinic Acetylcholine Receptor in the Rat Brain
Source: Mar Drugs. 2024 Apr 27;22(5):200. doi: 10.3390/md22050200 (PMC11122421; doi:10.3390/md22050200)
Supplement: Supplementary file 1 [file marinedrugs-22-00200-s001.zip › marinedrugs-2967692-supplementary.pdf]

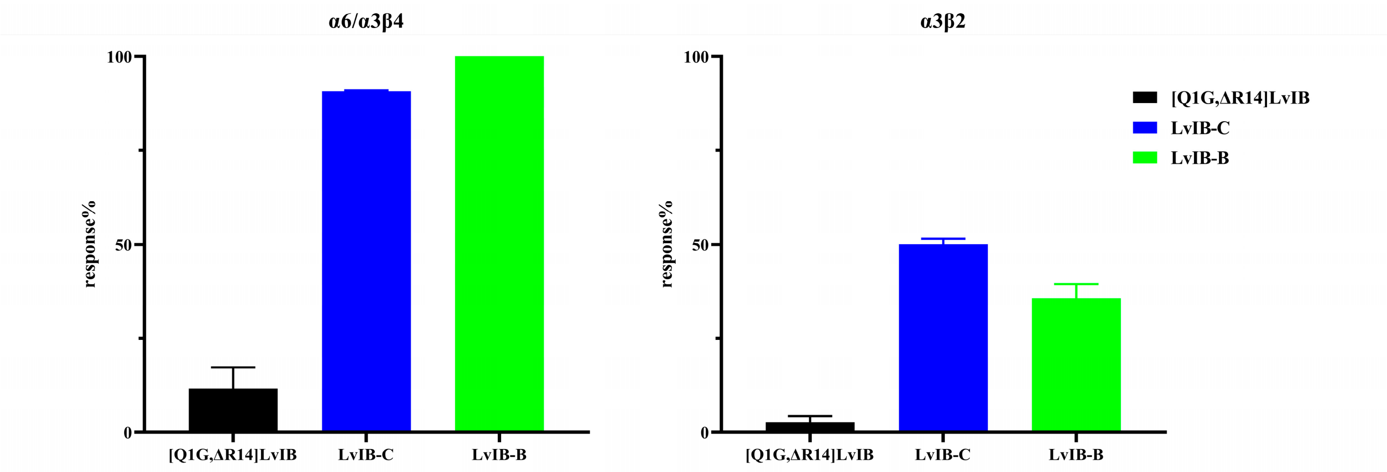

(A) (B)

Figure S1 Potency of [Q1G, ΔR14]LvIB, LvIB-C, and LvIB-B against other nAChR subtypes. At a concentration of 10 μM, both LvIB-C and LvIB-B showed a significant decrease in activity against *α6/α3β4* (A) and *α3β2* (B) nAChRs relative to the native peptide. The data was obtained from three individual oocytes.

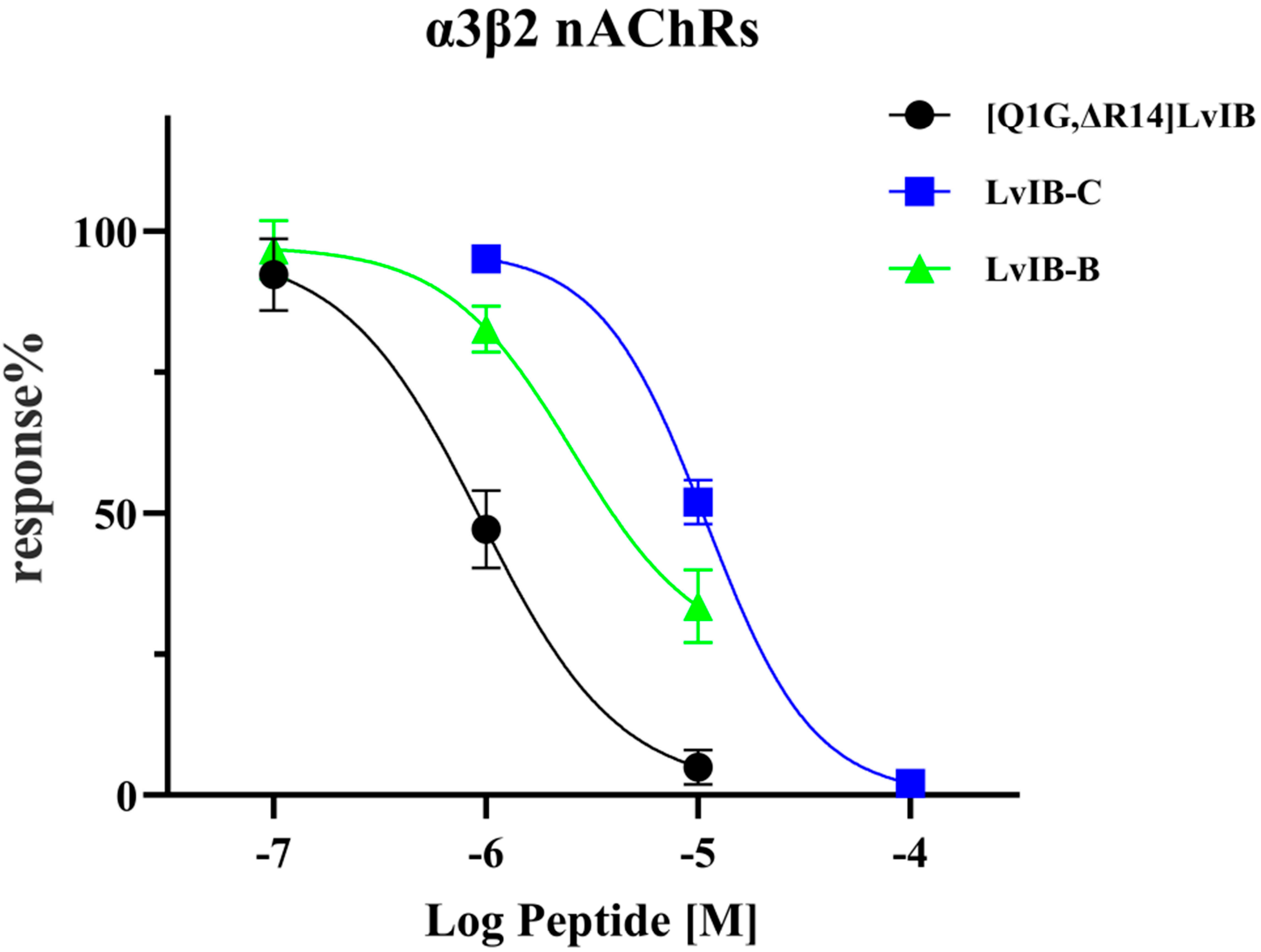

Figure S2 Concentration-response analysis of [Q1G, ΔR14]LvIB and its fluorescent analogs against *α3β2* nAChRs. The results showed that the IC_{50} value of [Q1G, ΔR14]LvIB, LvIB-C, and LvIB-B was 945 nM, 10.92 μM, and 2.55 μM, respectively. All data indicated as Mean±SEM and obtained from at least 3 independent oocytes.

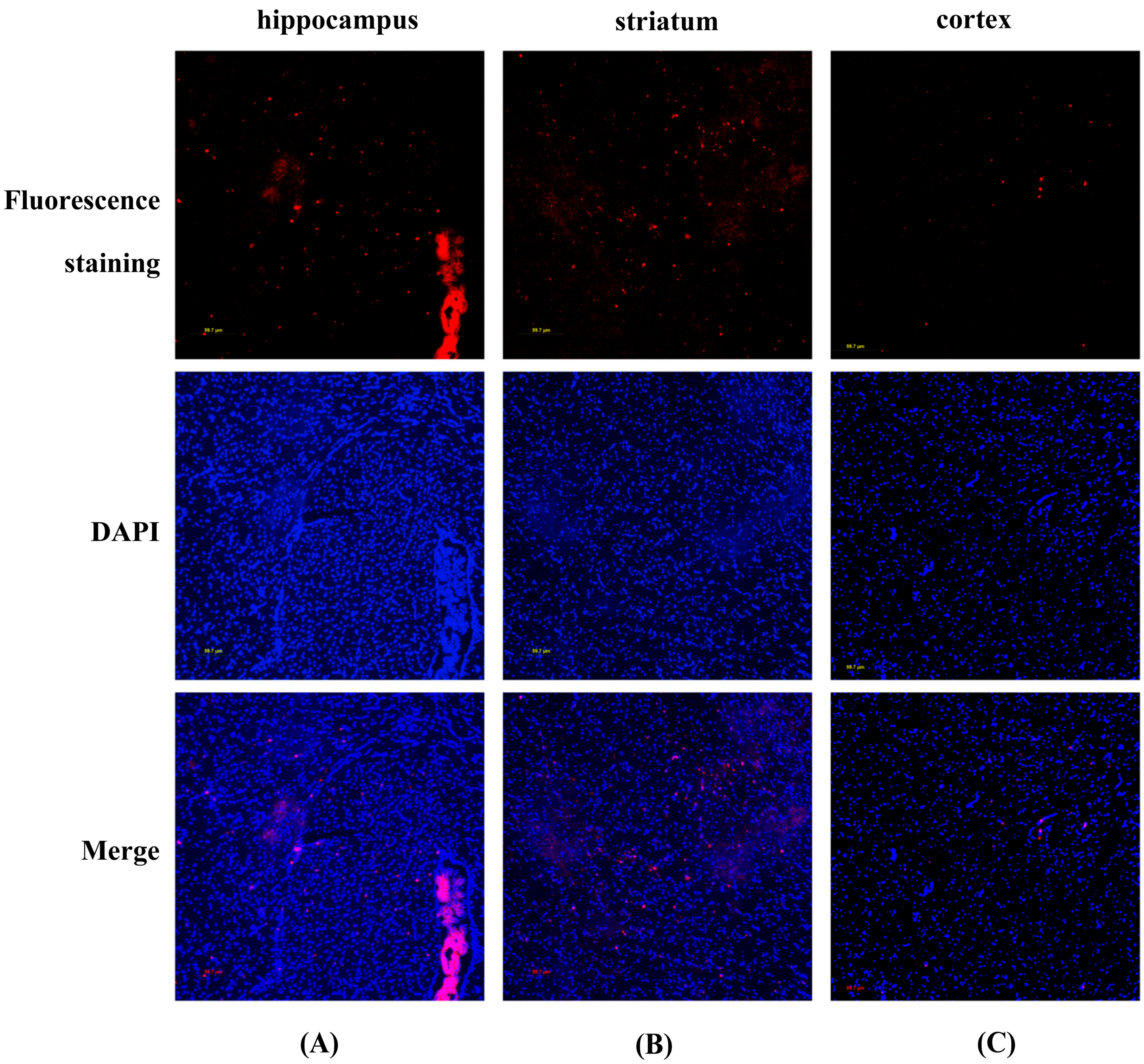

Figure S3 LvIB-R showed obvious fluorescence distribution in the hippocampus, striatum, and cortex of rat brain slices. The top row is red fluorescence emitted by LvIB-R, the middle row is blue fluorescence emitted by DAPI nuclear staining, and the bottom row is the mergence of red and blue fluorescence. (A) The hippocampus region was labeled with a large number of receptors by LvIB-R and exhibits red fluorescence. (B) Large amounts of red fluorescence were also seen in the striatum region. (C) A small amount of red fluorescence was seen in the cortex region. So we infer that *α7* nAChR is less distributed in the cortex than in the hippocampus and striatum regions.

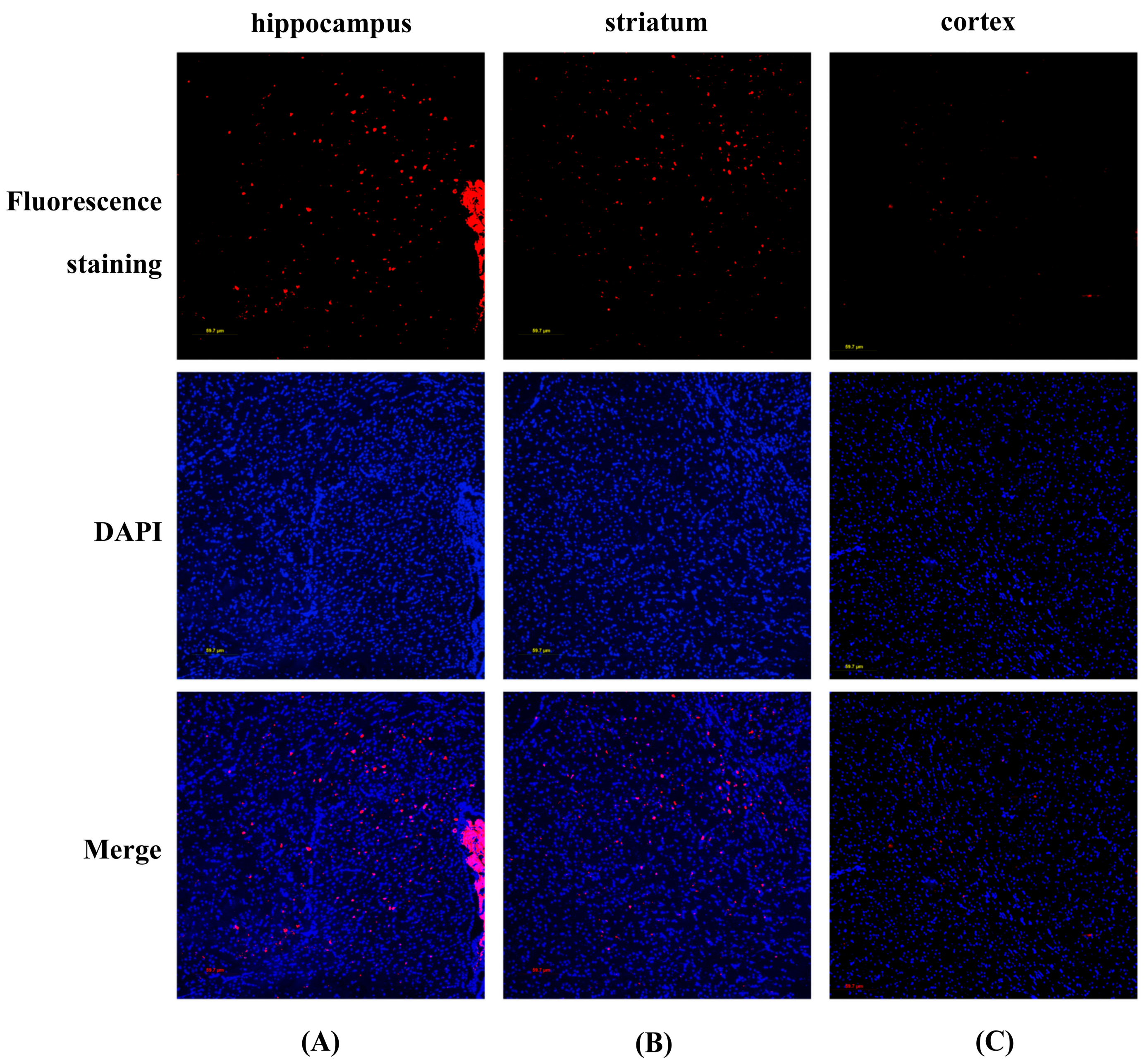

Figure S4 LvIB-C showed obvious fluorescence distribution in the hippocampus, striatum, and cerebral cortex of rats. The top row is the red fluorescence emitted by LvIB-C, the middle row is the blue fluorescence emitted by DAPI nuclear staining, and the bottom row is the mergence of red and blue fluorescence. (A) Large amounts of red fluorescence were detected by LvIB-C in the hippocampus. (B) Large amounts of red fluorescence were also observed in the striatum region, but less than the hippocampus region. (C) The cortex showed a little red fluorescence, and the amount of labeled $\alpha 7$ nAChR was less than that in the hippocampus and striatum regions.

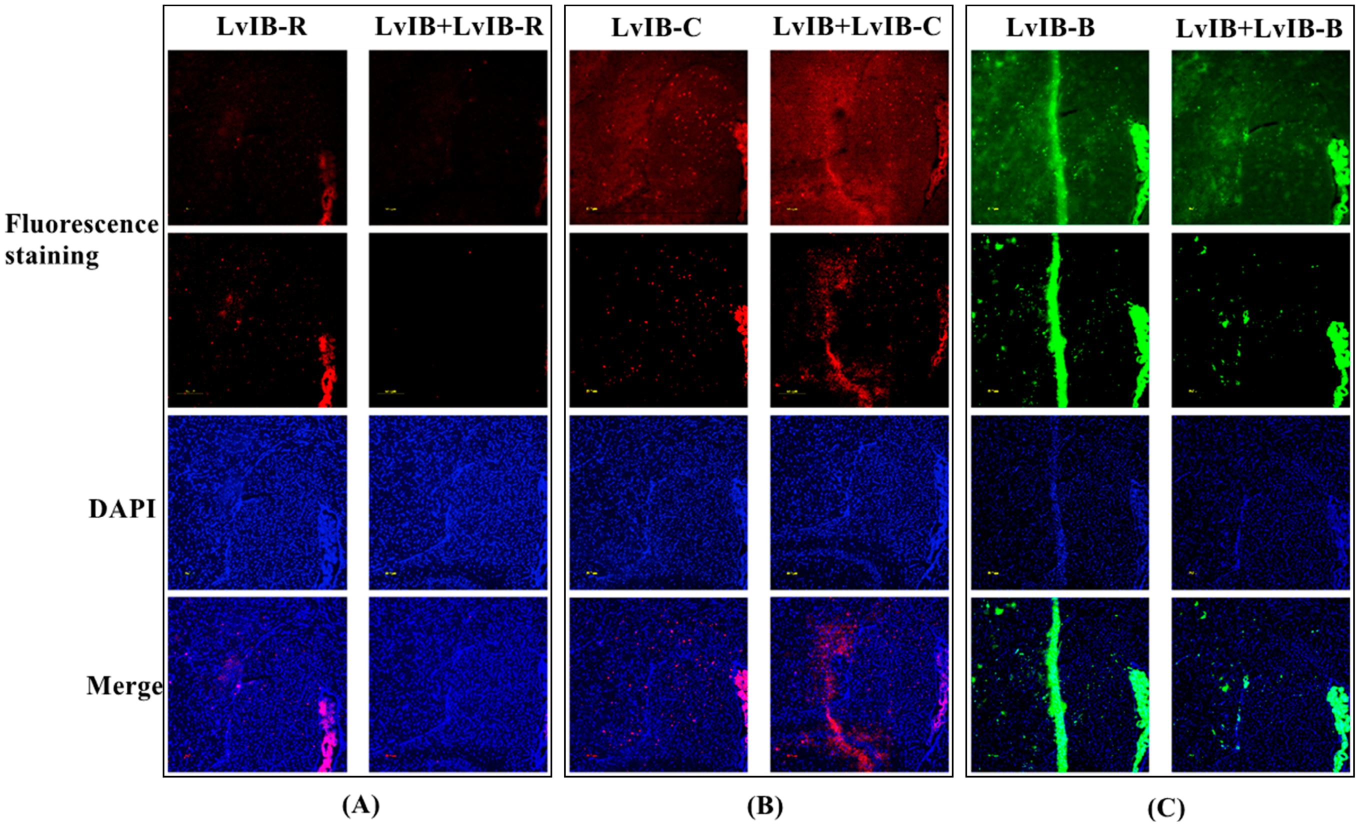

Figure S5 Competitive inhibition of *α7* nAChR when 1 μM concentration of native peptide and fluorescent analogs were co-incubated with the hippocampus of rat brain slices. (A) There was a definite decrease in red fluorescent spots after co-incubation with LvIB-R and [Q1G, ΔR14]LvIB. (B) The slices were incubated with LvIB-C and native peptide at the same time, and the marked *α7* nAChR decreased. (C) More green fluorescent spots were marked by LvIB-B.

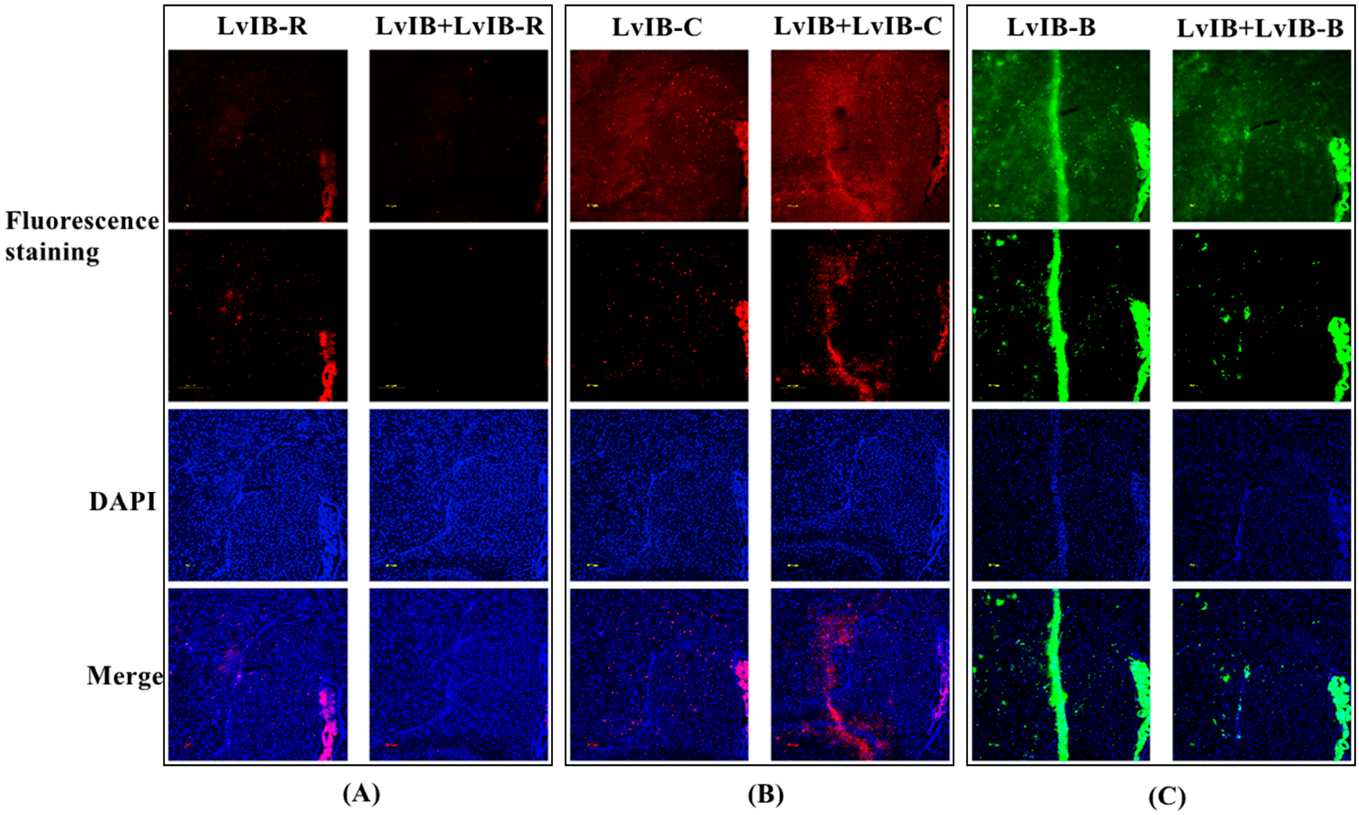

Figure S6 Fluorescence of [Q1G, ΔR14]LvIB and fluorescent analogs at 1 μM concentration in the striatal region of rat brain slices. (A) After co-incubation with LvIB-R and native peptide, the red fluorescent spots were reduced. (B) LvIB-C was incubated with native peptide at the same time, and the *α7* nAChR labeled was reduced. (C) There was a decrease in the number of green fluorescent dots labeled with LvIB-B.

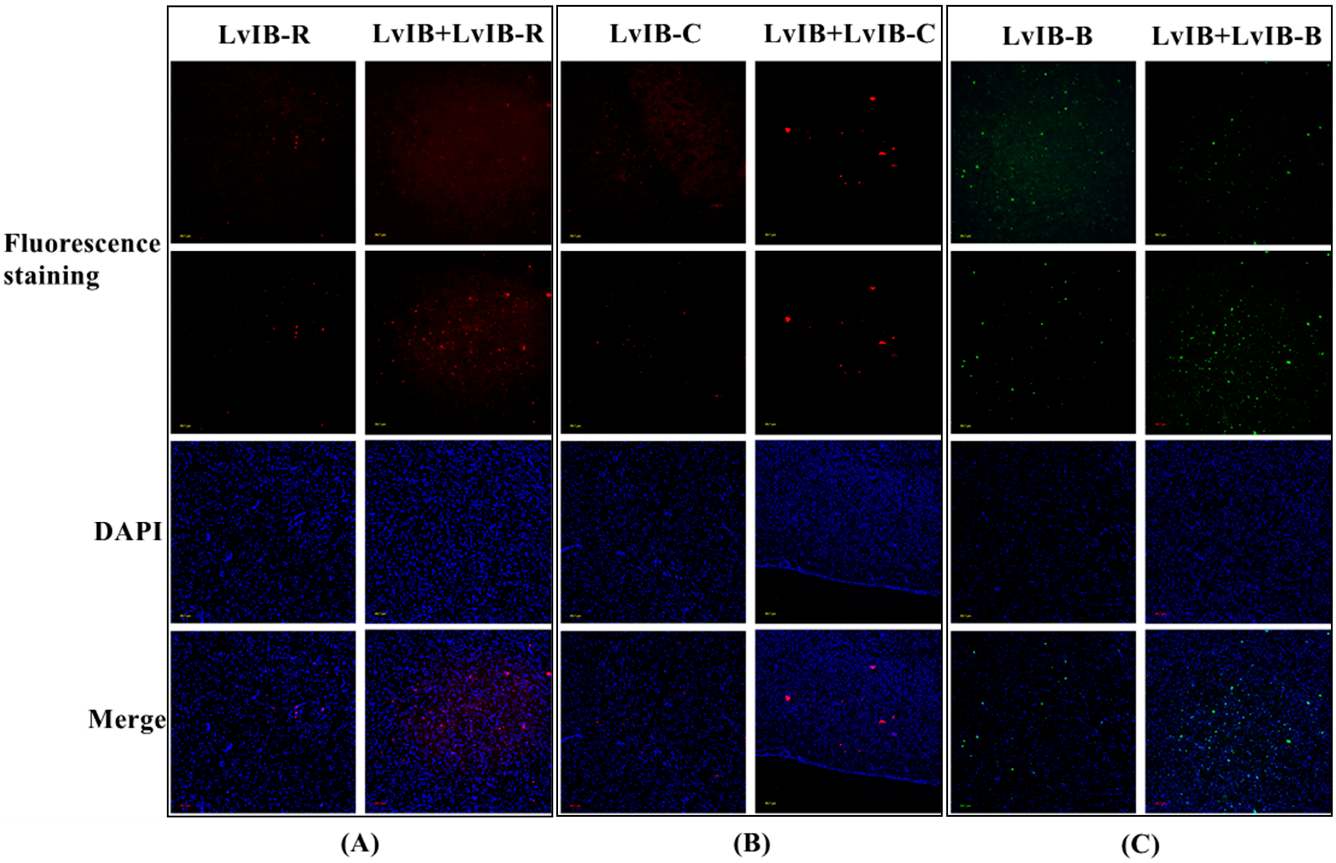

Figure S7 Fluorescence images of the cortex region in rat brain slices showing the competitive inhibition effect of native peptide and fluorescent analogous at 1 μM concentration. (A) Co-incubation of LvIB-R and [Q1G, ΔR14]LvIB with brain slices resulted in a decrease of red fluorescence spots. (B) Reduction in red fluorescence spots labeled by LvIB-C which targets *α7* nAChR. (C) Competitive binding of the native peptide with *α7* nAChR, leads to the decrease of green fluorescence spots labeled by LvIB-B.

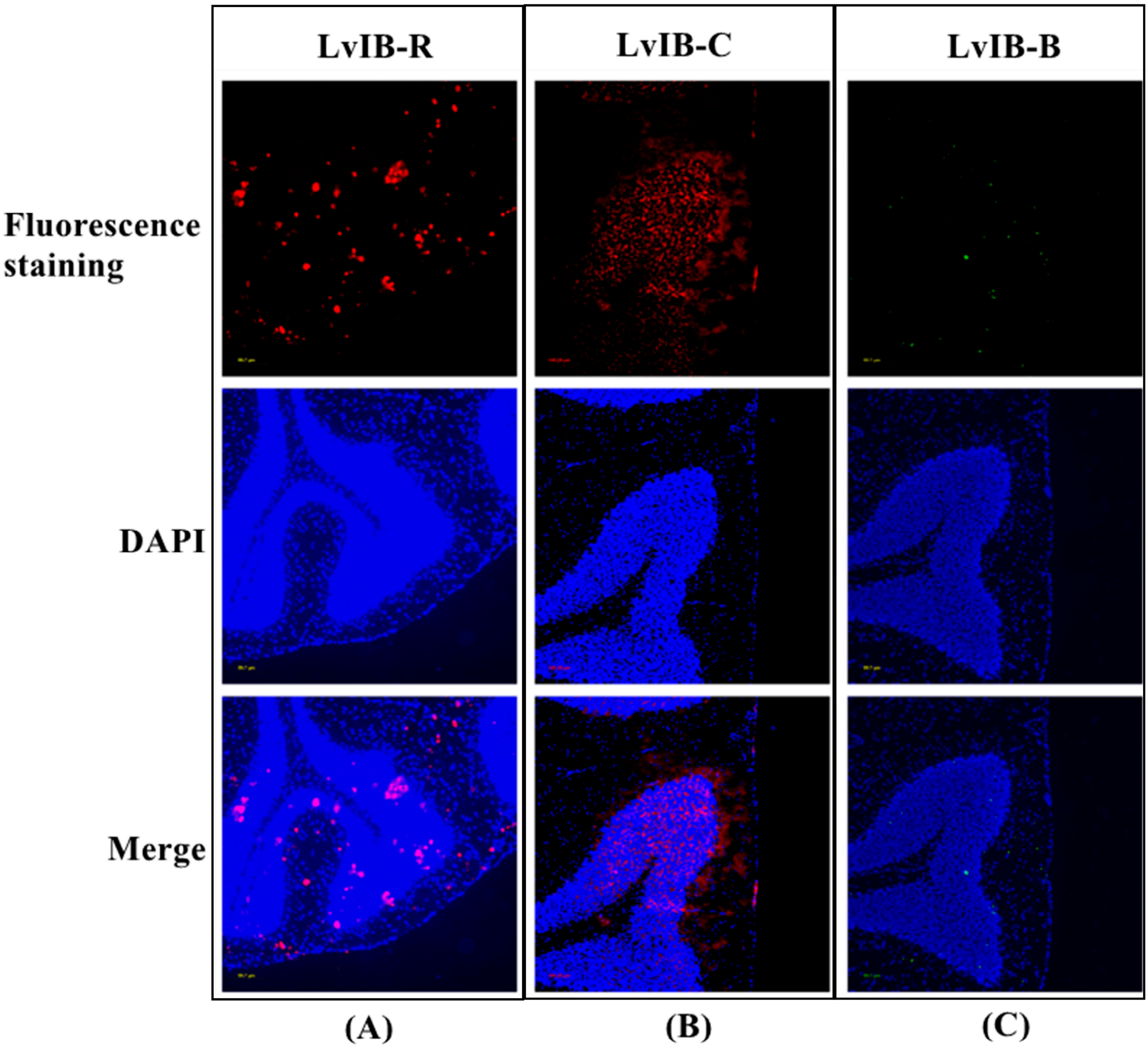

Figure S8 Different staining effects of three fluorescent peptides in the cerebellar region. Some *α7* nAChR can be labeled by fluorescent analogs and can be used as pharmacological tools. (A) *α7* nAChR in cerebellar regions labeled with LvIB-R shows red fluorescence. (B) Red fluorescence of LvIB-C in the cerebellar region. (C) Partial green fluorescent spots in cerebellar regions in which LvIB-B is labeled.
